# Mixed toxin profiles in two new and one known species of *Inocybe (Inocybaceae)* from northern China

**DOI:** 10.3897/mycokeys.141.203741

**Published:** 2026-09-29

**Authors:** Jia-Qi Liang, Xin Chen, Tie-Zhi Liu, Yi-Zhe Zhang, Cheng-Ye Sun, Zhong-Feng Li, Jia-Ju Zhong, Hai-Jiao Li, Yu-Guang Fan, Fei Xu

**Affiliations:** 1 Center for Disease Control and Prevention of Yiyang, 413000, Yiyang, China Center for Disease Control and Prevention of Yiyang Yiyang China; 2 State Key Laboratory of Trauma and Chemical Poisoning, National Institute of Occupational Health and Poison Control, Chinese Center for Disease Control and Prevention, 100050, Beijing, China State Key Laboratory of Trauma and Chemical Poisoning, National Institute of Occupational Health and Poison Control, Chinese Center for Disease Control and Prevention Beijing China; 3 School of Pharmacy, Hainan Medical University, 571199, Haikou, China School of Pharmacy, Hainan Medical University Haikou China; 4 College of Chemistry and Life Sciences, Chifeng University, 024005, Chifeng, China College of Chemistry and Life Sciences, Chifeng University Chifeng China; 5 Physical and Chemical Department, Ningxia Hui Autonomous Region Center for Disease Control and Prevention, 750000, Yinchuan, China Physical and Chemical Department, Ningxia Hui Autonomous Region Center for Disease Control and Prevention Yinchuan China

**Keywords:** LC-MS/MS, muscarine, new species, poisonous mushroom, psilocybin

## Abstract

Mushroom poisoning has become a serious food safety issue in China. Poisoning incidents caused by species of *Inocybaceae* have been continuously reported from tropical to temperate regions of China in recent years. Many species of *Inocybaceae* contain muscarine, which stimulates the parasympathetic nervous system; some also contain psilocybin, resulting in hallucinations, and a few even contain amatoxins. In this study, two new species of the *I. aeruginascens* group, *Inocybe tumericolor* and *I. subaeruginascens*, are described based on combined morphological evidence and multilocus phylogenetic analyses (ITS, LSU, and *rpb2*). Subsequently, a targeted multi-toxin analysis using liquid chromatography-tandem mass spectrometry (LC-MS/MS) was performed on these two new species together with the north-temperate species *I. asterospora*, quantifying 12 toxins, including muscarine, two isoxazole derivatives, three tryptamine alkaloids, three amatoxins, and three phallotoxins. Mixed profiles of muscarine, psilocybin, and psilocin were uncovered, whereas the other nine toxins were below the detection limits in all tested samples. *Inocybe tumericolor* contained psilocybin (1692.2–2758.7 mg/kg) and psilocin (77.8–134.8 mg/kg) but no muscarine; *I. asterospora* contained muscarine (79.6–1182.6 mg/kg) and psilocybin (107.1–765.2 mg/kg) but no psilocin; whereas *I. subaeruginascens* contained all three: muscarine (480.8–752.6 mg/kg), psilocybin (1023.0–2731.2 mg/kg), and psilocin (71.7–148.4 mg/kg). Detailed morphological characters and phylogenetic analyses of the two new species are also provided. These results may enrich knowledge of the diversity of *Inocybe* species and their toxins and provide scientific evidence for elucidating toxin evolution and facilitating the early diagnosis and treatment of mushroom poisoning. The presence or absence of preformed psilocin may affect the onset of hallucinogenic symptoms, underscoring the need for species-level identification in poisoning management.

## Introduction

Mushroom poisoning has become a leading cause of foodborne outbreaks and outbreak-associated deaths in China (Li et al. 2020). The latest study reported 1303 patients and 16 deaths from 505 mushroom poisoning incidents involving 97 different poisonous mushroom species in China in 2023 (Li et al. 2024). Currently, 660 poisonous mushroom species have been reported in China, and approximately 220 species are responsible for mushroom poisoning (Chen et al. 2016; Wu et al. 2019; Li et al. 2020, 2021a, 2022a, 2022b, 2023, 2024; Bau et al. 2024). Among these poisonous mushrooms, poisonous species of *Inocybaceae* constitute an important and species-rich group, mainly containing muscarine, which stimulates the parasympathetic nervous system (Li et al. 2020, 2021a, 2022a, 2023, 2024, 2025).

*Inocybe* (Fr.) Fr. was originally described as a group within *Agaricus* L. and later established as an independent genus in 1863 (Fries 1863). Recent phylogenetic studies have recognized *Inocybaceae* Jülich as a distinct family (Matheny et al. 2020). Currently, *Inocybaceae* (sensu lato) comprises seven genera, with *Inocybe* sensu stricto representing the largest genus (Matheny et al. 2020). As of 2025, approximately 140 species of *Inocybe* have been reported from China (Fan and Bau 2010, 2013, 2014a, 2014b, 2017, 2020; Fan et al. 2013, 2018; Bau and Fan 2018; Deng et al. 2021; Li et al. 2021b, 2022b; Chen et al. 2024; Gao et al. 2025; Zhu et al. 2025), and approximately 23 species of *Inocybe* s. str. are considered toxic (Mao 2006; Bau et al. 2014; Xu et al. 2020; Li et al. 2021a). Nonetheless, the species diversity of toxic *Inocybe* taxa in China remains understudied (Xu et al. 2020; Li et al. 2023, 2024).

Many species of *Inocybaceae* contain muscarine and stimulate the parasympathetic nervous system, and some species also produce psilocybin, ibotenic acid, muscimol, and phalloidin (Chen et al. 2016; Patocka et al. 2021; Li et al. 2022b; Zhang et al. 2022). Muscarine is the primary toxic metabolite in *Inocybe*, inducing a rapid onset (15 min–2 h) of classic parasympathetic stimulation; clinical manifestations comprise the triad of increased sweating, salivation, and lacrimation (White et al. 2019). Psilocybin causes rapid effects, with signs and symptoms manifesting 10–30 min after ingestion (more rapidly if taken in commercial liquid form). These include central nervous system (CNS) effects, illusions (visual, auditory, or tactile), true hallucinations (<50% of cases), an altered sense of time and space, synesthesia, and feelings of euphoria, often described as a mystical experience that may be long-lasting (Griffiths et al. 2006, 2008, 2011; Chen et al. 2016; White et al. 2019).

As part of a systematic toxin screening survey across a broad taxonomic sampling of the family *Inocybaceae* in northern China, unusual neurotoxin profiles were unexpectedly discovered in three representative species of *Inocybe*. These included two undescribed species belonging to the *I. aeruginascens* group, as well as the morphologically distinct *I. asterospora*. Different toxin combinations were detected among all three species. Based on integrative evidence from morphology, molecular phylogeny, and toxin profiling, the two new species are formally described and illustrated in this study.

## Materials and methods

### Morphological studies

Specimens were collected from Beijing, Jilin, and Nei Mongol in northern China. Fresh basidiomata were photographed *in situ*, and measurements of pileus diameter, lamellae width, stipe length and width, and context thickness were taken shortly after collection. All collected samples were dried at 45 °C with an electronic dryer overnight, and all studied specimens were processed and deposited in the National Institute of Occupational Health and Poison Control, Chinese Center for Disease Control (**NIOHP**, **China CDC**), the Herbarium of Changbai Mountain Nature Reserve (**ANTU**, with FCAS numbers), and the Mycological Herbarium of Chifeng University (**CFSZ**). Macromorphological descriptions were based on field notes and color photographs of basidiomata. Color codes followed Kornerup and Wanscher (1978). The microscopic procedure followed Yu et al. (2020). In the description of the basidiospores, the abbreviation [*n*/*m*/*p*] means *n* basidiospores measured from *m* basidiomata of *p* collections; dimensions of basidiospores are given using the following notation: (a–) b–c (–d). The range ‘b–c’ contains a minimum of 90% of the measured values; extreme values (a, d) are given in parentheses. *L* means spore length (arithmetic average of all spores). *W* means spore width (arithmetic average of all spores). *Q* means the length/width ratio of a spore in side view; *Q_m_* means the average *Q* of all basidiospores measured ± sample standard deviation.

### DNA extraction and sequencing

The Phire® Plant Direct PCR Kit (Finnzymes Oy, Finland) was used to obtain PCR products from dried specimens according to the manufacturer’s instructions, with some modifications (Tang et al. 2023). The primer pairs ITS5/ITS4, LR0R/LR7, and bRPB2-6F/bRPB2-7.1R were used to amplify the internal transcribed spacer (ITS) region, nuclear ribosomal large subunit (nrLSU), and *rpb2*, respectively (White et al. 1990; Vilgalys and Hester 1990; Matheny 2005). The PCR cycling conditions followed Wang et al. (2021). The PCR products were then sent to the Beijing Genomics Institute for purification and sequencing.

### Phylogenetic analyses

A total of 22 sequences (12 ITS, five nrLSU, and five *rpb2*) were obtained and submitted to GenBank. The sequences obtained in this study were compared with those available in the GenBank database (https://www.ncbi.nlm.nih.gov/) for phylogenetic analyses. Species belonging to *Inocybe* sect. *Lactiferae*, sect. *Hysterices*, and the *I. asterospora* group were selected based on a literature survey (Matheny and Kudzma 2019; Zhao et al. 2022). The three single-gene datasets were aligned using the MAFFT online service (Katoh et al. 2019). The alignments were manually adjusted using BioEdit version 7.0.9.0 (Hall 1999). The datasets of individual loci were concatenated using MEGA 5.02 (Tamura et al. 2011). *Mallocybe pallidipes* Y.G. Fan, J.H. Hu & W.J. Yu and *M. longicystis* T. Bau, Y.G. Fan, J.H. Hu & W.J. Yu were selected as outgroups (Hu et al. 2023). Maximum likelihood (ML) analyses were performed using the W-IQ-TREE web service (http://iqtree.cibiv.univie.ac.at/) with 1000 ultrafast bootstrap replicates (Trifinopoulos et al. 2016). Bayesian inference (BI) analyses were performed in MrBayes v.3.2.7a (Ronquist et al. 2012); the best-fit models were selected using MrModeltest v2.3 (Nylander 2004).

### Toxin detection

The extraction and purification methods were based on previously published methods (Xu et al. 2020; Zhang et al. 2022) and are briefly described as follows. Dried mushroom powder (10 mg) and 2 mL of methanol/water (70:30, v/v) were added to the same 15 mL centrifuge tube. Subsequently, the tube was placed in an ultrasonic bath for 1 h, vortexed for 30 s, and centrifuged at 15,000 rpm for 5 min at 4 °C. The supernatant was purified using a QuCHERS-PP column (Huaren Health Company, Henan, China) to remove impurities such as pigments and lipids. An aliquot of the cleanup solution was transferred into the sample bottle and diluted to 1 mL with ultrapure water obtained using a Milli-Q water purification system (Millipore, Milford, MA, USA) before analysis using a 4000 QTrap LC-MS/MS system (AB Sciex, Foster City, CA, USA).

The optimal LC-MS/MS detection parameters and conditions were based on previously published methods (Xu et al. 2020; Zhang et al. 2022). The 12 main mushroom toxins (muscarine, two isoxazole derivatives, three tryptamine alkaloids, three amatoxins, and three phallotoxins) (Alta Scientific Co., Ltd., Tianjin, China) were detected qualitatively and quantitatively using LC-MS/MS. The performance of the method was validated according to Regulation (EU) 2021/808 (EU 2021). *Lentinula edodes* was used as a sample blank for determining the limits of detection (LOD; *S*/*N* = 3), limits of quantitation (LOQ; *S*/*N* = 10), accuracy, and precision in this study.

## Results

### Molecular phylogeny

The final multilocus dataset (Table 1) comprised 72 taxa and 2740 characters, including an 830-bp ITS region, a 1200-bp nrLSU region, and a 710-bp *rpb2* gene. The alignment was deposited in TreeBase under accession number 31316. For the BI analysis, the following substitution models were selected for each data partition using the AIC in MrModeltest v2.3: GTR+I+G for ITS, LSU, and *rpb2*. The BI phylogenetic analysis was stopped after 705,000 generations, when the average standard deviation of split frequencies converged to 0.009696, the effective sample size was 1,479.35, and the average potential scale reduction factor parameter value was 1.001. The following models selected using the BIC were used for the ML analysis, which yielded a final log-likelihood value of −15901.58: TVM+F+I+G4 for ITS and TN+F+I++G4 for LSU and *rpb2*.

**Table 1.** Information of taxa used in the phylogenetic analysis.

**Taxa**	**Collection no**.	**Country**	**GenBank accession numbers**		
			**ITS**	**28S**	***rpb2***
*Inocybe aeruginascens*	JG270502(TENN063863)	Germany	GU949590	JN974970	–
*I. aeruginascens*	JG310508 (TENN063936)	Germany	GU949591	MH220256	MH249787
*I. aeruginascens*	PC111007 (TENN063908)	South Africa	GU949592	MH220257	–
*I. asterospora*	EL372-19	France	OP001731	OP001731	–
*I. asterospora*	EL100-14		MN296110	MN296110	–
*I. cf. asterospora*	MR00015	Sweden	AM882897	AM882897	–
*I. asterospora*	170727-12	China	PP574574	–	–
*I. asterospora*	BJMTG20230905-09	China	PP574575	PP573135	–
*I. asterospora*	20210806-34	China	PP574576	–	–
*I. asterospora*	20210806-38	China	PP574577	–	–
*I. chondroderma*	PBM1760 (WTU)	British Columbia	GU949586	MH220258	–
*I. chondroderma*	PBM1776 (WTU)	Washington	GU949579	JN974967	MH249789
*I. corydalina*	AM10687	Russia	MH216083	MH220259	MH249790
*I. corydalina*	EL124/04	Sweden	AM882736	AM882736	–
*I. corydalina*	TURA 6488 (WTU)	Belgium	–	AY038314	AY337370
*I. tumericolor*	FYG8483	China	PZ269976	PZ269997	PZ276333
*I. tumericolor*	FYG8484 (type)	China	PZ269977	PZ269998	PZ276334
*I. tumericolor*	C1 (CFSZ 22566)	China	PZ269970	–	–
*I. tumericolor*	C13 (CFSZ 22578)	China	PZ269971	–	–
*I. tumericolor*	C22 (CFSZ 22587)	China	PZ269969	–	–
*I. tumericolor*	A86 (CFSZ 22446)	China	PZ269968	–	–
*I. dulciolens*	LVK13340	New Jersey	MH216084	MH220261	MH249792
*I. dulciolens*	PBM2646 (holotype, TENN062477)	Tennessee	MH216088	MH220265	MH249796
*I. erinaceomorpha*	EL128/05	Sweden	AM882735	AM882735	–
*I. erinaceomorpha*	JV14756F (TURA7645)	Sweden	MH216089	MH220266	MH249797
*I. fraudans*	AMB 12793 (epitype)	Italy	KM873363	–	–
*I. fraudans*	EL11604	Sweden	AM882731	AM882731	–
*I. fraudans*	EL12304	Sweden	AM882729	–	–
*I. fraudans*	EL15105	Sweden	AM882730	AM882730	
*I. fraudans*	TF01-037	Denmark	AJ889953	–	–
*I. cf. fraudans*	KBE13496	Sweden	FN550914	FN550914	–
*I. glabripes*	JV7318F (TURA3804)	Finland	MH216096	–	MH249803
*I. haemacta*	AMB21462	Italy	JF908180	–	–
*I. hystrix*	HRL11842	Quebec	KX897428	–	–
*I. hystrix*	SJ020824	Sweden	AM882810	AM882810	–
*I. hystrix*	PBM3300 (TENN064304)	North Carolina	GU949588	MH220275	–
*I. hystrix*	RS31493 (WTU)	Finland	–	AY380380	AY337381
*I. aff. hystrix*	REH7405 (NY)	Costa Rica	GU949589	JN974969	MH249806
*I. melanopus*	BJ920904	Sweden	AM882725	AM882725	–
*I. melanopus*	JV4986	Finland	AM882727	AM882727	–
*I. melanopus*	PBM3975 (TENN068973)	Tennessee	–	MH220276	MH249807
*I. melanopus*	Stz3641 (holotype, WTU)	Washington	–	HQ201359	–
*I. melanopus*	TAA185135	Estonia	AM882726	–	–
*I. moelleri*	EL68/07	Sweden	FN550913	FN550913	–
*I. moelleri*	SJ88062	Sweden	AM882737	AM882737	–
*I. meirensongia*	FYG3777 (type)	China	MZ322672	MZ322669	MZ336924
*I. meirensongia*	FGY3776	China	MZ322671	–	–
*I. meirensongia*	FYG3552	China	PZ269975	–	–
*I. meirensongia*	FYG9686	China	PZ269972	PZ269995	PZ276332
*I. meirensongia*	FYG9678	China	PZ269973	–	–
*I. meirensongia*	FYG9683	China	PZ269974	–	–
*I. aff. pallidobrunnea*	PBM1957 (WTU)	Washington	MH216098	MH220277	MH249808
*I. aff. pallidobrunnea*	PBM2242 (WTU)	Washington	MH216099	JN974968	MH249809
*I. phari*	SMNS-STU-F-0901774 (holotype)	Friesland	OQ324787	–	–
*I. pileosulcata*	DED8058 (SFSU, holotype)	Thailand	EU600838	EU600838	–
I. pyriodora var. chamaesalicis	EL64/05	Norway	AM882733	AM882733	–
*I. rekhankitha*	CAL1356 (holotype)	India	KY440102	KY549132	–
*I. subaeruginascens*	FYG9883 (holotype)	China	PP574571	PP573132	PP583565
*I. subaeruginascens*	FYG9884	China	PP574572	PP573133	PP583566
*I. subaeruginascens*	FYG9885	China	PP574573	PP573134	PP583567
*I. subaeruginascens*	H37 (CFSZ 24139)	China	PP573137	–	–
*I. subaeruginascens*	K30 (CFSZ 23464)	China	PP573138	–	–
*I. subaeruginascens*	I94 (CFSZ 24345)	China	PP573139	–	–
*Inocybe* sp.	KB04072008 (TENN 063524)	California	MH216103	JN974963	MH249812
*Inocybe* sp.	*Quercus* ECM bg8ae5	California	EF559275	–	–
*Inocybe* sp.	src156	California	DQ974804	–	–
*I. tricolor*	SJ05011	Sweden	AM882738	AM882738	–
*Mallocybe longicystis*	FYG6374	China	OM179928	OM135611	OM835747
*M. pallidipes*	FYG3726	China	OM179924	OM137052	OM835749
*Nothocybe distincta*	ZT 9250	India	–	EU604546	EU600904
*Pseudosperma fulvidiscum*	FYG6311 (holotype)	China	OM135592	OM349998	OM747849
*P. fulvidiscum*	FYG6058	China	OM135593	OM349999	OM780121
*P. singulare*	FYG6339	China	OM135605	OM149380	OM780122
*P. singulare*	FYG6363 (holotype)	China	OM135606	OM149381	OM780123
*P. singulare*	FYG6365	China	OM135607	OM149382	OM780124

Notes: Samples collected in the present study are marked in bold.

**Figure 1.**
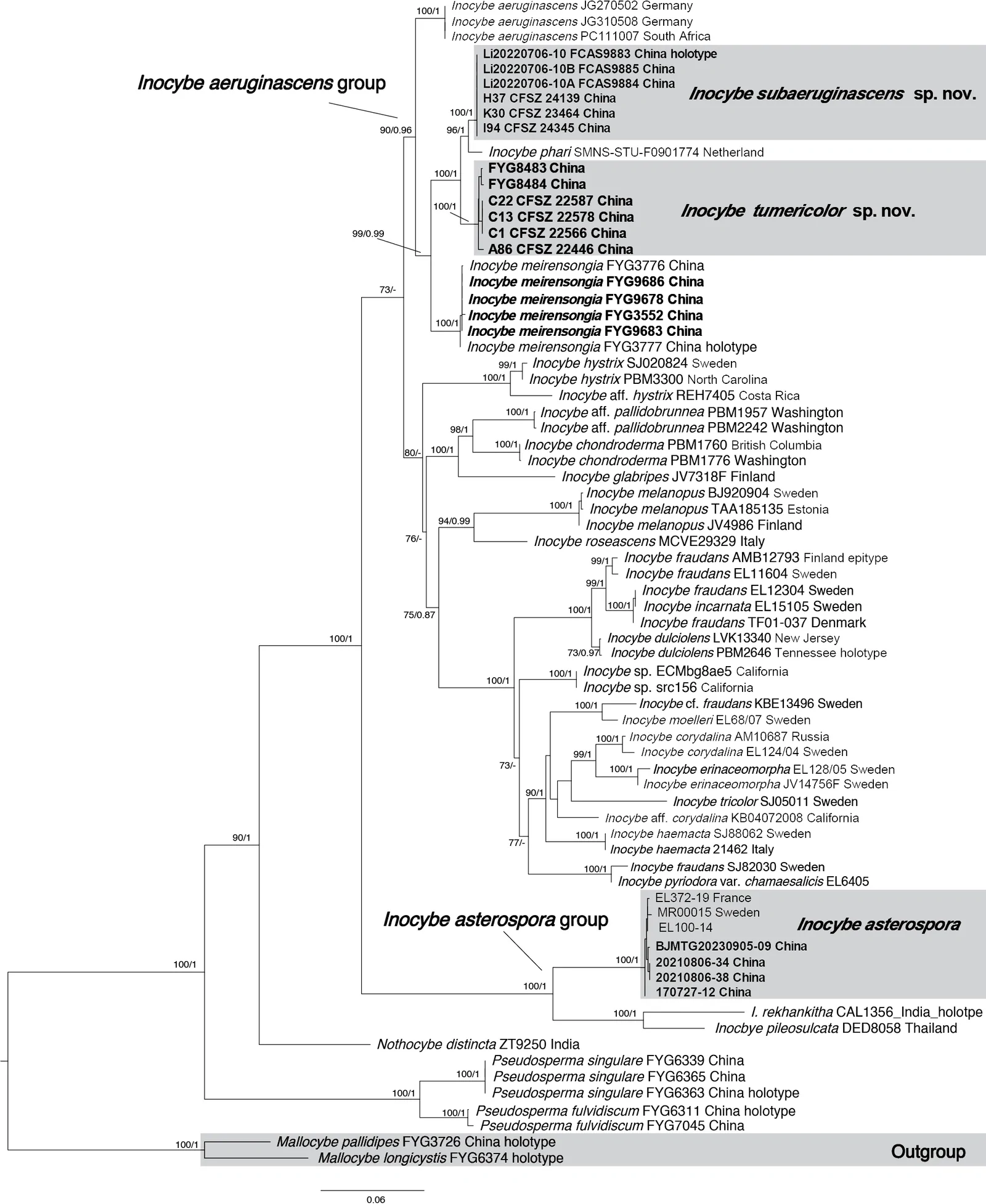
Phylogram generated by Bayesian analysis based on sequences from the combined dataset (ITS, nrLSU, and *rpb2*). Bayesian inference posterior probabilities (BI-PP) ≥ 0.95 and ML bootstrap proportions (ML-BP) ≥ 70 are represented as BI-PP/ML-BP.

The best-scoring trees generated from ML and BI analyses are shown in Fig. 1. The ingroup received full support (BP/PP = 100/1.00) and was resolved into three distinct genera, namely *Inocybe*, *Nothocybe*, and *Pseudosperma*. Four specimens (170727-12, BJMTG20230905-09, 20210806-34, and 20210806-38) clustered with authentic reference materials of *I. asterospora* (EL100-14 and EL372-19) within a fully supported clade (BP/PP = 100/1.00). Six specimens representing the proposed new species *I. subaeruginascens* formed a well-supported independent clade (BP/PP = 100/1). Similarly, six specimens of the other proposed new species, *I. tumericolor*, grouped into a fully supported clade (BP/PP = 100/1). *Inocybe subaeruginascens* and *I. phari* formed sister clades, and this combined lineage was sister to the *I. tumericolor* clade.

### Taxonomy

#### Inocybe tumericolor

Taxon classificationFungiAgaricalesInocybaceae

Y.G. Fan, T.Z. Liu, Hai J. Li, F. Xu & X. Chen sp. nov.

56C7E777-93E2-56EA-86CC-9A725B631BA1

864079

Figs 2, 3

##### Chinese name.

姜黄丝盖伞 (Jiāng Huáng Sī Gài Săn).

**Figure 2.**
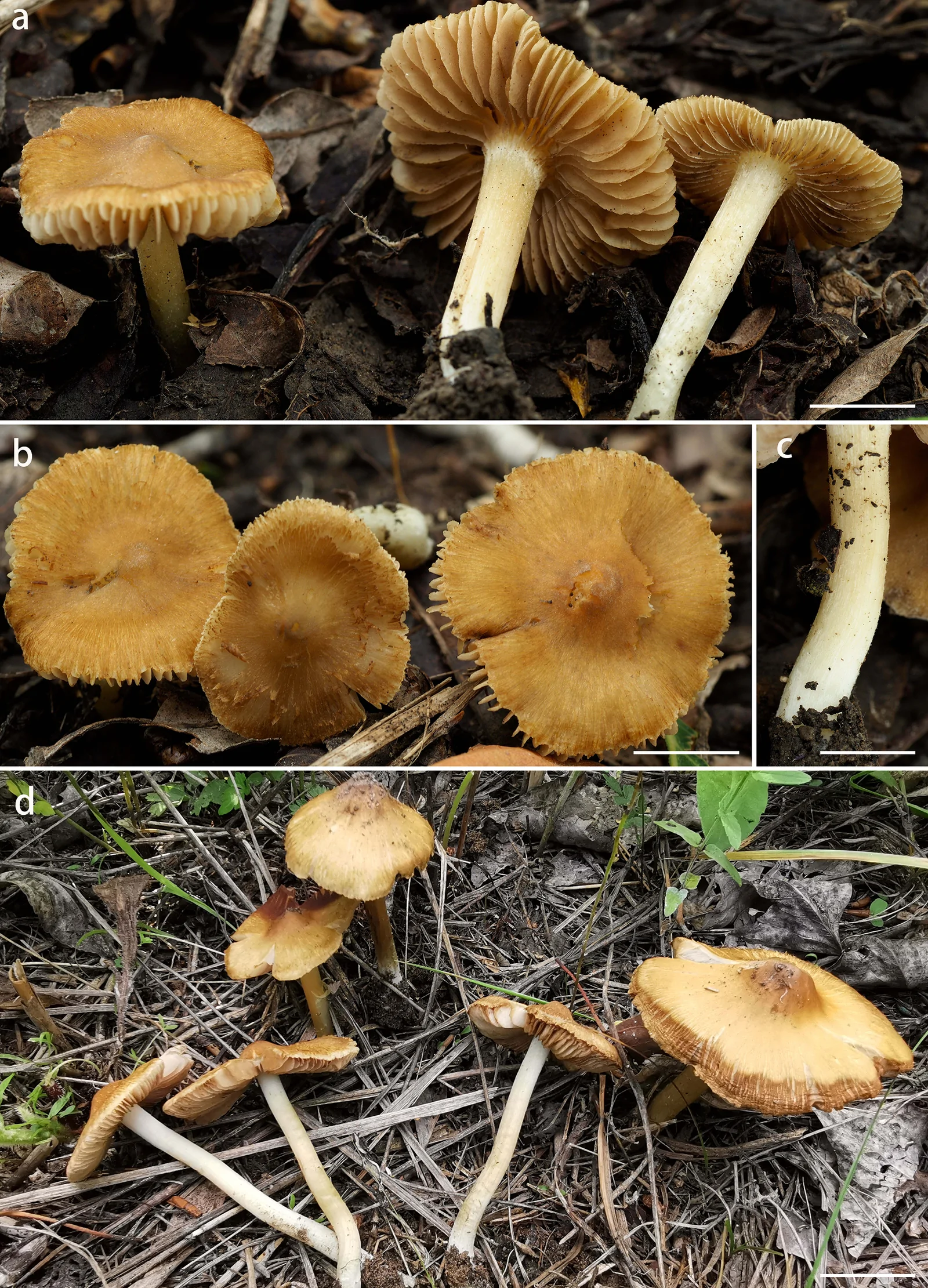
Basidiomata of *Inocybe tumericolor*. **a–c**. FYG8484 (FCAS4284, holotype); **d**. CFSZ 22466. Scale bars: 1 cm.

**Figure 3.**
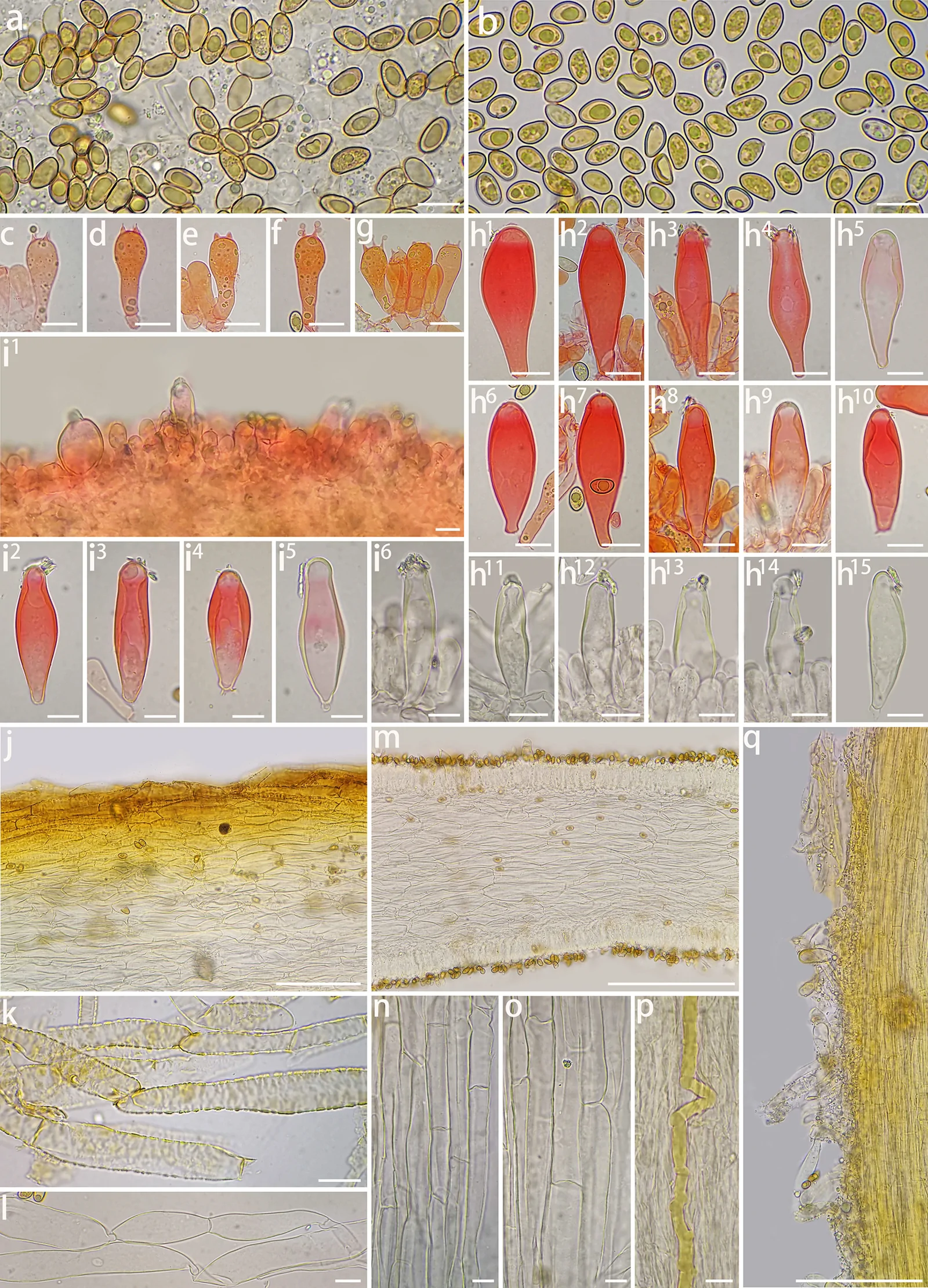
Microscopic features of *Inocybe tumericolor* FYG8484 (FCAS4284, holotype). **a, b**. Basidiospores; **c–g**. Basidia; **h^1^–h^15^**. Pleurocystidia; **i^1^–i^6^**. Cheilocystidia; **j**. Pileipellis; **k**. Encrusted pileipellis hyphae; **l**. Pileal trama hyphae; **m**. Cross section of lamellae; **n**. Stipitipellis and stipitipellis hyphae; **o**. Stipe trama hyphae; **p**. Oleiferous hyphae. Photos by Xin Chen. Scale bars: 10 μm (**a–i, j–l, n–p**); 100 μm (**j, m, q**).

##### Etymology.

Referring to the turmeric-yellow color of the pileus.

##### Holotype.

China • Jilin Province: Changchun City, Nong’an County, in artificial plantations of *Populus alba* × *P. berolinensis*, 2 Aug. 2023, Y.G. Fan & W.J. Yu, FYG8484 (FCAS4441).

##### Diagnosis.

The new species is distinguished by a glabrous, warm yellow pileus, a whitish stipe, and lamellae that typically exceed the pileus margin. It resembles *I. subaeruginascens* but differs in having a stipe without blue tinges, shorter hymenial cystidia, and 4% ITS sequence divergence.

##### Description.

Basidiomata small-sized. Pileus 20–30 mm diam, hemispherical to plano-convex when young to mature, broadly umbonate, margin entire to crenulated, initially incurved to uplifted when mature; surface dry, silky smooth when young, becoming fibrillose to cracked-fibrillose, but never rimulose; color uniformly warm yellow (4A7) or ginger yellow (4A8). Lamellae 0.5–1 mm wide, adnexed to decurrent, moderately crowded, ventricose, margin fibriate to undulate, unequal in length, alternately distributed with 3–4 tiers of lamellulae, pallid when young, pale yellowish (2A2) to yellowish brown (3A4) upon maturity. Stipe 30–36 × 3–3.5 mm, terete, equal, solid, slightly swollen at the apex and base; whitish (2A1) flocculose towards the apices, pale yellowish (3A3) at the middle to apex, base white (2A1). Context fleshy in pileus, 0.5 mm thick at mid-radius, up to 3 mm thick under the umbo, white (2A1) to ivory white (2A2), pale yellowish brown near the apex; fibrillose in the stipe, whitish (2A1). Odor fungoid.

Basidiospores (5.6) 7.5–8.8–9.2 (9.9) × (3.5) 4.5–5.1–5.5 (6.0) μm (*n* = 100/2/2), *Q* = (1.14)1.50–1.66–1.87(2.02), *Q_m_* ± SD = 1.66 ± 0.16, smooth, ellipsoid or amygdaliform, apiculus distinct, with intracellular circular to oblong oil inclusions, pale yellow to light yellow. Basidia 24–32 × 7–12 μm, mostly claviform to broadly claviform, hyaline, 4-spored, sterigmata 2–5 μm length. Pleurocystidia 43–51–60 × 10–15–20 μm (*n* = 40), mostly broadly fusiform or lageniform, at times ventricose to (sub) utriform, apices obtuse or rounded, crystallization present at the apices, base usually tapered into a small pedicel, at times obtuse and non-pedicellate towards the base, slightly thick-walled, walls up to 1 μm thick, strikingly thickened at the middle (up to 3 μm), pale yellow-greenish. Cheilocystidia 36–41–52 × 10–14–19 μm (*n* = 40), resemble pleurocystidia, mostly fusiform and broadly fusiform, at times subcylindrical. Hymenophoral trama 75–130 μm thick, regularly arranged, colorless, hyphae, thin-walled, colorless, smooth, mostly slightly inflated hyphae 6–25 μm wide, cylindrical hyphae occasionally observed. Pileipellis a cutis, 75–100 μm wide, yellowish to yellowish brown in mass, composed of cylindrical and inflated (8–17 μm wide) and cylindrical hyphae (5–10 μm wide), thin-walled, and strongly encrusted. Pileal trama 350–610 μm thick, subregularly arranged, colorless, hyphae 13–25 μm wide, inflated, smooth, and thin-walled. Caulocystidia 35–45–70 × 11–15–21 μm, mostly fusiform and lageniform, thick-walled, walls pale yellow-greenish in 5% KOH, apex crystallization, descending the entire stipe length. Stipitipellis regularly arranged, pale yellow-greenish in mass, composed of cylindrical hyphae, 4–18 μm wide, thin-walled, smooth. Oleiferous hyphae 2–6 µm wide, yellowish, diverticulate, mostly present in the stipe trama. Clamp connections present and common in all tissues.

##### Habitat and ecology.

Single or scattered in plantations of *Populus alba* × *P. berolinensis*.

##### Distribution.

China (Jilin and Nei Mongol Autonomous Region).

##### Additional specimens examined.

China • Jilin Province: Changchun City, Nong’an County, in artificial plantations of *Populus alba* × *P. berolinensis*, 2 Aug. 2023, Y.G. Fan & W.J. Yu, FYG8483 (FCAS4440); • Nei Mongol Autonomous Region, Ulanqab City, Sumu Mountain, Xinghe County, 14 July 2020, Liu Tiezhi, Yang Xiaopo, Song Delin, A86 (CFSZ 22446); • Xilingol League, Duolun County, Luanyuan Town, Dagushanzi Village, 15 July 2020, Liu Tiezhi, Yang Xiaopo, Song Delin, C1 (CFSZ 22566), C13 (CFSZ 22578), C22 (CFSZ 22587).

##### Remarks.

*Inocybe tumericolor* is an occasional species occurring from July to August in artificial plantations of *Populus alba* × *P. berolinensis* in northern China. The new species is characterized by the nearly glabrous, ginger-yellow pileus, a pallid to yellowish stipe, and moderately crowded lamellae that are usually longer than the pileus. Phylogenetically, *I. tumericolor* is sister to the lineage unifying *I. phari* and *I. subaeruginascens*. However, *I. phari*, described from Europe, has a brown to reddish-brown pileus, longer and narrower basidiospores (8.3–9.7–10.9 × 4.5–4.9–5.7 µm; *Q* = 1.7–2.0–2.3 [SD = 0.1]), an ecological association with *Salix*, and a 4.8% (32/670) sequence difference in the ITS region (Bandini et al. 2023). *Inocybe subaeruginascens*, described in the present study, has a similar outward appearance, but it has a dull yellow pileus, a blue-tinged stipe base, longer hymenial cystidia (up to 86 µm in length), and a 4% (27/670) sequence difference in the ITS region.

#### Inocybe subaeruginascens

Taxon classificationFungiAgaricalesInocybaceae

Y.G. Fan, Hai J. Li, F. Xu, T.Z. Liu & Jia-Qi Liang sp. nov.

3B50CF70-2DCD-5183-85D9-464BA7BD55F0

853495

Figs 4, 5

##### Chinese name.

青脚丝盖伞 (Qīng Jiăo Sī Gài Săn).

**Figure 4.**
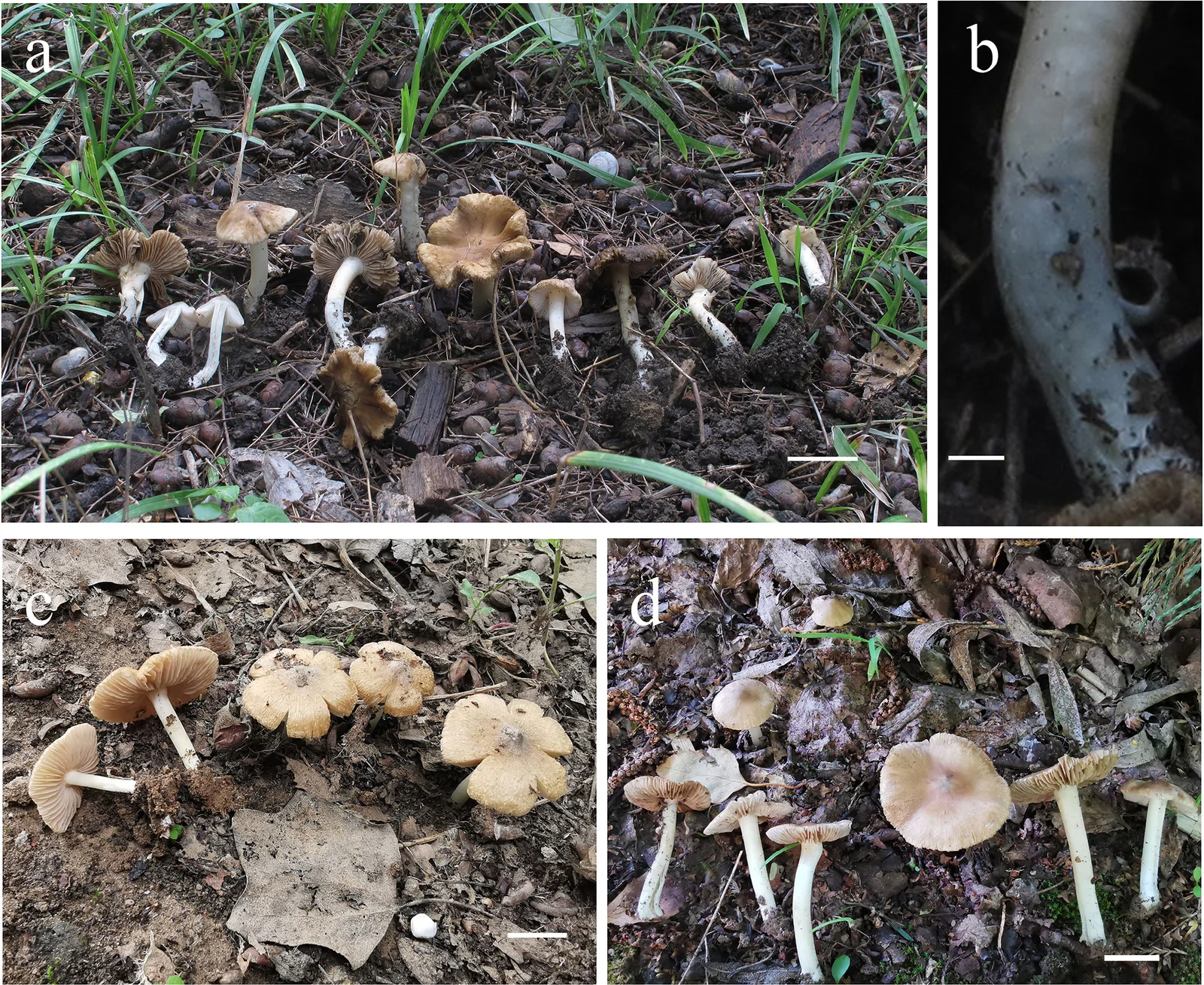
Basidiomata of *Inocybe subaeruginascens*. **a, b**. Li20220706-10 (FCAS9883, holotype); **c, d**. CFSZ 23464. Scale bars: 1 cm (**a, c**); 0.5 cm (**b**).

**Figure 5.**
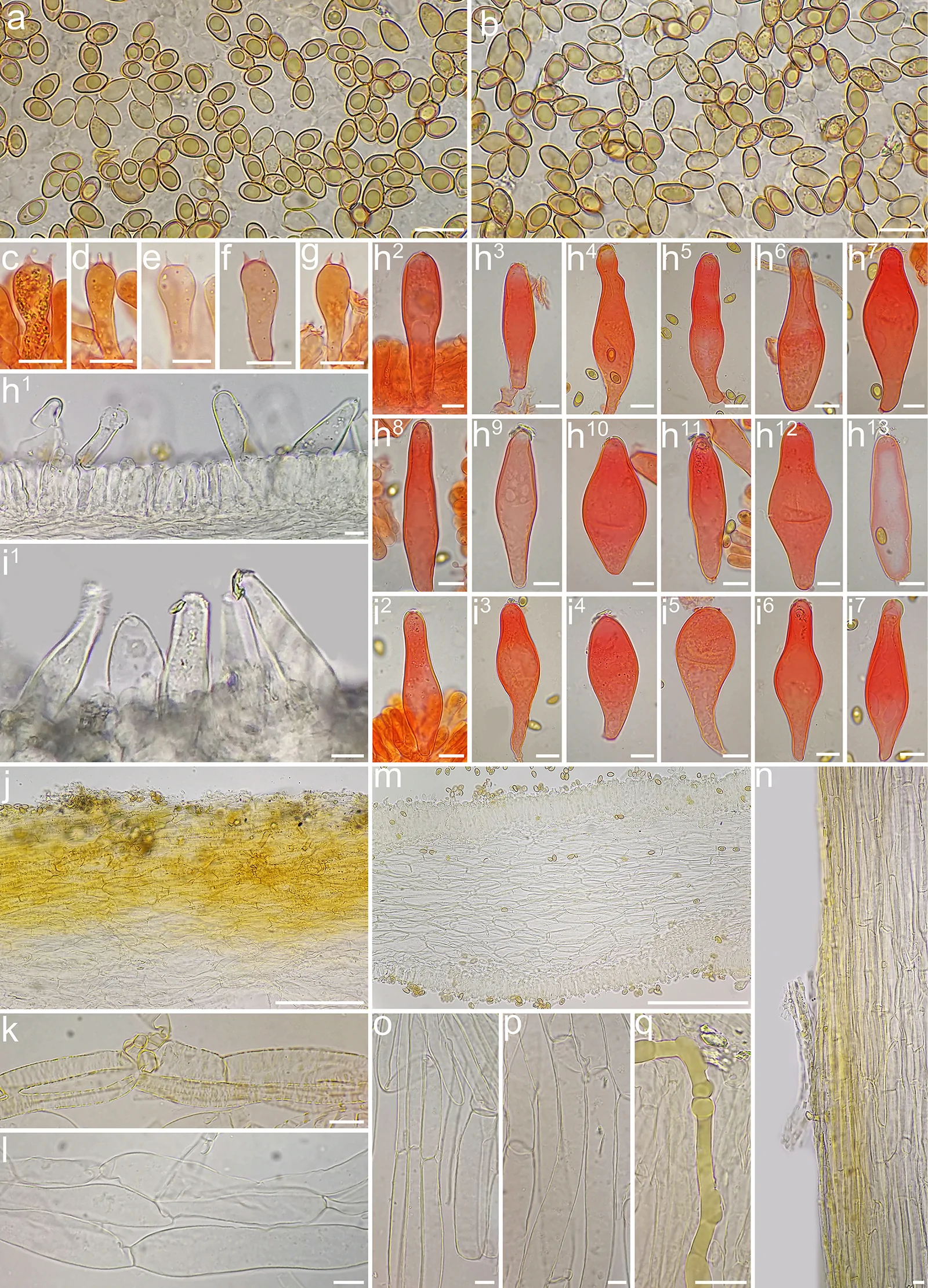
Microscopic features of *Inocybe subaeruginascens* (Li20220706-10, holotype). **a, b**. Basidiospores; **c–g**. Basidia; **h^1^–h^13^**. Pleurocystidia; **i^1^–i^7^**. Cheilocystidia; **j**. Pileipellis; **k**. Encrusted pileipellis hyphae; **l**. Pileal trama hyphae; **m**. Cross section of lamellae; **n, o**. Stipitipellis and stipitipellis hyphae; **p**. Oleiferous hyphae. Photos by Xin Chen. Scale bars: 10 μm.

##### Etymology.

*subaeruginascens*, in reference to the similar species *Inocybe aeruginascens*.

##### Holotype.

China • Beijing: Dongcheng district, Temple of Heaven, alt: 40 m, 39°53'3"N, 116°24'4"E, on the ground of *Sophora japonica*, 6 July 2022, Li20220706-10 (FCAS9883, holotype). GenBank accession numbers: ITS–PP574571, LSU–PP573132 and RPB2–PP583565.

##### Diagnosis.

Pileus ochraceous yellow, stipe white with a bluish tint; Basidiospores 7–9.5 × 4–6 μm, hymenial cystidia as metuloids, versiform, mostly lageniform to sublageniform. *Inocybe subaeruginascens* differs from *I. aeruginascens* by the lack of a greenish tinge in the disc and stipe, the presence of a bluish tint in the lower part of the stipe, a different phylogenetic placement and ecology.

##### Description.

Basidiomata small. Pileus 10–29 mm in diam., at first conical to conical-convex, becoming broadly convex to concave with age, margin initially deflexed, straight to uplifted when mature. Surface dry, glabrous-fibrillose to appressed fibrillose, occasionally appressed scaly with protruding fibrils toward the margin; uniformly straw yellow (4A6) to dingy fawn (4B8) around the disc, paler outwards. Stipe 30–75 × 4–7 mm, cylindrical or slightly swollen toward the apex, solid, central or eccentric, nearly smooth with sparse fibrils at the stipe apex; white (1A1) to cream white (1B2) but with a bluish tinge (24B3) in the lower half. Lamellae crowded, adnexed, often subsinuate; initially white (1A1), then yellowish white (5A3), turning to yellowish brown (5B5) when old, edge concolorous, even. Context fleshy in pileus, 1–2 mm thick, white (1A1) to cream white (1B2); fibrillose in stipe, striate, shiny, concolorous with the pileus. Odour fungoid.

Basidiospores [100/3/3] (6.9) 7.1–8.0–8.9 (9.2) × (4.0) 4.2–4.9–5.1 (5.5) μm, *Q* = (1.36)1.47–1.62–1.82(1.98), *Q_m_* ± SD = 1.62 ± 0.11, ellipsoid to subamygdaliform, sub-conical at the apex, thick-walled, smooth, yellowish to yellowish brown. Basidia 20–30 × 6–9 μm (*n* = 15), mostly ellipsoid, sometimes broadly clavate, obtuse at the apex, then somewhat tapered towards the base, 4-spored. Pleurocystidia 55–70–86 × 12–18–30 μm (*n* = 40), abundant, mostly broadly fusiform or lageniform, at times (sub) cylindrical to (sub) utriform, apices obtuse or rounded, crystallization present at the apices, base usually tapered into a small pedicel, at times obtuse and non-pedicellate towards the base, slightly thick-walled, walls up to 1 μm thick, strikingly thickened at the apex or at the middle (up to 3 μm), pale greenish. Cheilocystidia 50–65–86 × 11–17–26 μm (*n* = 40), resemble pleurocystidia, mostly fusiform and broadly fusiform, at times cylindrical or (sub) utriform, often obtuse at the apex, sometimes slightly tapered into capitate to sub-capitate, becoming somewhat narrow towards the base, thick-walled. Hymenophoral trama 72–115 μm thick, regular, colorless, composed of thin-walled, smooth, cylindric to often inflated hyphae 10–26 μm wide, usually slightly constricted between two connected cells. Pileipellis a cutis, 87–185 μm wide, yellowish to yellowish brown in mass, composed of cylindrical and inflated hyphae, cylindrical hyphae, 6–10 μm wide, and inflated hyphae, 12–18 μm wide, thin-walled, and rough. Pileal trama 210–450 μm thick, subregularly arranged, colorless, hyphae 5–20 μm wide, smooth, thin-walled, inflated. Caulocystidia 42–60–75 × 10–15–22 μm (*n* = 20), mostly fusiform and lageniform, thick-walled, walls pale greenish. Stipitipellis regularly arranged, pale greenish in mass, composed of cylindrical hyphae, 7–13 μm wide, thin-walled, smooth. Oleiferous hyphae 2–5 µm wide, yellowish, diverticulate, mostly present in the pileal and stipe trama. Clamp connections present and common in all tissues.

##### Habitat and ecology.

Gregarious in *Sophora japonica* Linn. or *Populus alba var. pyramidalis* Bunge forests.

##### Distribution.

China (Beijing, Nei Mongol Autonomous Region).

##### Additional specimens examined.

China • Beijing: Dongcheng district, Temple of Heaven, alt: 40 m, 39°53'3"N, 116°24'4"E, on the ground under *Sophora japonica*, 6 July 2022, Li20220706-10A (FCAS9884), Li20220706-10B (FCAS9885); • Nei Mongol Autonomous Region, Chifeng City, on the campus of Chifeng University, 24 September 2020, under *Populus alba var. pyramidalis* Bunge, T.Z. Liu, K30 (CFSZ 23464), • same locality, 13 August 2021, T.Z. Liu, H37 (CFSZ 24139), • same locality, 25 August 2021, T.Z. Liu, I94 (CFSZ 24345), • same locality, 3 July 2022, T.Z. Liu, D71, (CFSZ 24769), T.Z. Liu, U77 (CFSZ 24826).

##### Remarks.

In the field, *I. subaeruginascens* can be easily recognized by its gregarious habit, small- to medium-sized basidiomata, straw-yellow pileus, and nearly smooth, white stipe with a bluish tinge in the lower part. The bluish tinge in the stipe could be overlooked without careful observation. Microscopically, it can be distinguished by ellipsoid to subamygdaliform basidiospores and versiform, extremely abundant, thick-walled hymenial cystidia ranging from subfusiform to sublageniform.

In the multigene phylogeny, *I. phari* Bandini & G. Bandini, recently described from Europe, is a sister to the present new species. These two species share ellipsoid to subamygdaliform basidiospores and slightly thick-walled hymenial cystidia. However, *I. phari* has a chestnut pileus, a pruinose stipe apex, narrower basidiospores measuring 8.3–10.9 × 4.5–5.7 µm, subutriform to subclavate often with no neck or a short neck, and an ecology in sandy dunes with *Salix* (Bandini et al. 2023). Moreover, a comparison of ITS sequence data reveals a 3% (19/630) difference between these two species. *Inocybe tumericolor*, described in the present study, has a similar outward appearance, but it has a ginger-yellow pileus, a non-blue-tinged stipe, shorter hymenial cystidia (<60 µm), and a 4% (27/670) sequence difference in the ITS region. *Inocybe meirensongia* Y.G. Fan, W.J. Yu & L.S. Deng, a recently described species from northeastern China, is macroscopically similar to the new species, but the former has pinkish stipes without any bluish tint and an ecology with *Pinus sylvestriformis* (Takenouchi) W.C. Cheng & C.D. Chu (Bhunjun et al. 2022). *Inocybe aeruginascens* Babos, another phylogenetically related species, is similar in having a yellow pileus and whitish stipes with a bluish-green tint in the lower part, but it has larger basidiospores measuring 8–10 × 4.5–5.7 µm and associations with *Populus*, *Salix*, or *Picea* (Kuyper 1986; Ludwig 2017).

### Toxin detection

**Figure 6.**
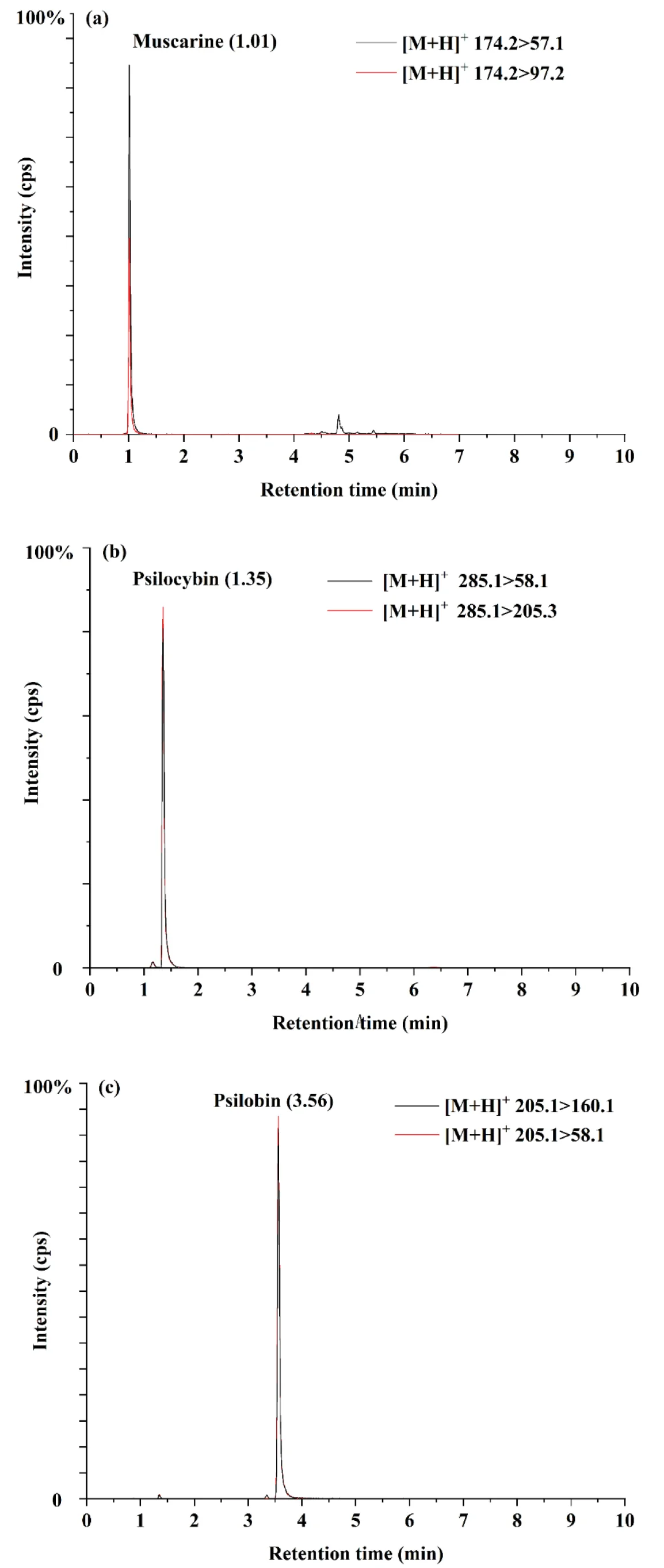
Multiple reaction monitoring (MRM) chromatograms of muscarine (**a**), psilocybin (**b**), and psilocin (**c**) in the collected mushroom samples.

In the qualitative analysis, three neurotoxins, muscarine, psilocybin, and muscimol, were detected in the collected samples, whereas nine other toxins were not detected (Fig. 6). For quantitative analysis, the LODs for muscarine, psilocybin, and psilocin in the matrix blank sample solution were 0.05, 0.1, and 0.05 mg/kg, respectively, and the LOQs were 0.2, 0.4, and 0.2 mg/kg, respectively. The three detected substances exhibited good linearity, with *R^2^* > 0.9940. The recoveries obtained for muscarine, psilocybin, and muscimol in spiked *L. edodes* mushrooms were 88.2–96.4%, 90.9–95.1%, and 93.1–96.1%, with relative standard deviations (RSDs) of 6.4–7.8%, 5.3–8.6%, and 5.5–9.3%, respectively, indicating good performance of the extraction and purification method used in this study. The quantitative results are shown in Table 2; all three species contained neurotoxins. In the known species *I. asterospora*, the contents of muscarine and psilocybin ranged from 79.6 to 1182.6 mg/kg and from 107.1 to 765.2 mg/kg, respectively, whereas psilocin was not detected. Regarding the two new species, *I. tumericolor* contained psilocybin (1692.2–2758.7 mg/kg) and psilocin (77.8–134.8 mg/kg) but no detectable muscarine. *Inocybe subaeruginascens* exhibited a mixed profile of all three toxins: muscarine (480.8–752.6 mg/kg), psilocybin (1023.0–2731.2 mg/kg), and psilocin (71.7–148.4 mg/kg).

**Table 2.** Muscarine, psilocybin, and psilocin concentrations in different specimens.

**Species**	**Voucher**	**Muscarine (mg/kg**)	**Psilocybin (mg/kg)**	**Psilocin (mg/kg)**	**Collecting Sites**	**Collecting Time**
*Inocybe asterospora*	170727-12	229.7	218.3	ND	Huairou district, Beijing	27 July 2017
*I. asterospora*	20210806-34	79.6	330.7	ND	Huairou district, Beijing	6 August 2021
*I. asterospora*	20210806-38	234.0	107.1	ND	Huairou district, Beijing	6 August 2021
*I. asterospora*	BJMTG20230905-09	1182.6	765.2	ND	Mentougou district, Beijing	5 September 2023
*I. subaeruginascens*	20220706-10	480.8	2219.3	107.6	Dongcheng district, Beijing	6 July 2022
*I. subaeruginascens*	CFSZ 23464	627.4	1023.0	73.5	Chifeng City, Nei Mongol	24 September 2020
*I. subaeruginascens*	CFSZ 24345	536.4	1023.0	71.7	Chifeng City, Nei Mongol	25 August 2021
*I. subaeruginascens*	CFSZ 24769	506.7	2731.2	114.9	Chifeng City, Nei Mongol	3 July 2022
*I. subaeruginascens*	CFSZ 24826	752.6	2643.7	148.4	Chifeng City, Nei Mongol	3 July 2022
*I. tumericolor*	CFSZ 22446	ND	1692.2	77.8	Ulanqab City, Nei Mongol	14 July 2020
*I. tumericolor*	CFSZ22566	ND	2739.3	97.9	Ulanqab City, Nei Mongol	15 July 2020
*I. tumericolor*	CFSZ 22578	ND	1908.3	127.1	Ulanqab City, Nei Mongol	15 July 2020
*I. tumericolor*	CFSZ22587	ND	2758.7	134.8	Ulanqab City, Nei Mongol	15 July 2020

ND: not detected (<LOD).

## Discussion

Over the past 5 years, 15 species belonging to the family *Inocybaceae*, including *Inocybe amelandica* Bandini & B. Oertel, *I. aff. ericetorum*, *I. aff. glabrodisca*, *I. serotina* Peck, *Inosperma hainanense* Y.G. Fan, L.S. Deng, W.J. Yu & N.K. Zeng, *In. muscarium* Y.G. Fan, L.S. Deng, W.J. Yu & N.K. Zeng, *In. aff. virosum*, *In. cf. virosum*, *Mallocybe fulvipes* (Kühner) Matheny & Esteve-Rav., *M. fulvoumbonata* (Murrill) Matheny & Esteve-Rav., *Pseudosperma arenarium* Y.G. Fan, Fei Xu, Hai J. Li & Vauras, *P. conviviale* Cervini, Bizio & P. Alvarado, *P. triaciculare* Saba & Khalid, *P. umbrinellum* (Bres.) Matheny & Esteve-Rav., and *P. yunnanense* (T. Bau & Y.G. Fan) Matheny & Esteve-Rav., have been identified from mushroom poisoning incidents across China (Li et al. 2020, 2021a, 2022a, 2023, 2024, 2025). Additionally, several other toxic species of *Inocybaceae* have been uncovered through toxin screening in recent years (Xu et al. 2020; Li et al. 2021b; Mao et al. 2022; Zhang et al. 2022; Zhao et al. 2022). The present study further clarifies species diversity and toxin profiles within the genus *Inocybe* by describing two new species, *I. tumericolor* and *I. subaeruginascens*, and documenting novel mixed profiles of muscarine, psilocybin, and psilocin in representative taxa. These findings provide primary data for public education, risk prevention, and clinical diagnosis and treatment of mushroom poisoning.

Previous studies reported five European species of *Inocybe* to be psilocybin-positive: *I. aeruginascens*, *I. coelestium*, *I. corydalina*, *I. haemacta*, and *I. tricolor*. All five belong to the smooth-spored temperate clade, exhibit bluish or reddish coloration, and are devoid of muscarine. Kosentka et al. (2013) further supported the notion that the evolutionary loss of muscarine biosynthesis preceded the acquisition of psilocybin production, even though not all muscarine-deficient taxa are capable of synthesizing psilocybin. However, the discovery of *I. subaeruginascens* and the stellate-spored *I. asterospora*, which produce both psilocybin and muscarine, challenges this previously inferred evolutionary link between muscarine loss and psilocybin gain. Meanwhile, the detection of psilocybin in *I. asterospora* represents the first report of this toxin in a nodulose-spored lineage, expanding the phylogenetic distribution of psilocybin within the genus *Inocybe*. These findings imply that the evolutionary dynamics underlying secondary metabolite production in *Inocybe* are more intricate than previously assumed.

Psilocybin is a prodrug that requires dephosphorylation to generate the active metabolite psilocin, which directly acts on 5-HT2A receptors, producing psychoactive effects and neuropsychiatric symptoms (e.g., hallucinations and disorientation) lasting 4–6 hours, with most symptoms resolving within 24 hours (Dinis-Oliveira 2017). In the toxin profiling, psilocin was consistently detected in *I. subaeruginascens* (71.7–148.4 mg/kg) and *I. tumericolor* (77.8–134.8 mg/kg), which lacks muscarine, whereas it was absent in all examined *I. asterospora* specimens despite their high psilocybin content (up to 765 mg/kg; Table 2). This indicates that the capacity to dephosphorylate Ethicalpsilocybin to psilocin is not universal among psilocybin-producing *Inocybe* species. The presence of preformed psilocin may lead to a more rapid onset of hallucinogenic effects by bypassing the first-pass dephosphorylation step. Accordingly, this may result in different clinical presentations, underscoring the need for species-level identification in both poisoning surveillance and clinical management of *Inocybe*-related mushroom poisoning in China.

Currently, the *Inocybe aeruginascens* group includes five formally recognized species. Previous studies have reported the presence of psilocybin but not muscarine in *I. aeruginascens*, a toxin profile similar to that of the new species *I. tumericolor* described herein. However, toxin profiling has not been performed for either the European species *I. phari* or the northern Chinese species *I. meirensongia*, owing to limited available material. Consequently, the full toxin spectrum across the *I. aeruginascens* group remains incompletely understood. Several limitations should be acknowledged in the present study. Toxin profiling for *I. meirensongia* and *I. phari* was not possible because of limited material, and the small sample size for certain species may not fully capture intraspecific variation in toxin composition. Moreover, the genetic basis underlying the interspecific differences in dephosphorylation capacity was not investigated. Future studies should undertake comprehensive toxin characterization and quantification—including simultaneous measurement of muscarine, psilocybin, and psilocin—for additional members of both the *I. aeruginascens* group and the *I. asterospora* group to better elucidate the evolutionary trajectory and biogeographic patterns of toxin biosynthesis in these fungi.

In summary, the findings reveal that the evolutionary transition between muscarine and psilocybin in *Inocybe* is not unidirectional and that preformed psilocin varies markedly among species, with direct implications for clinical toxidromes. Accurate species identification and toxin profiling are therefore essential for risk assessment and patient management.

## Supplementary Material

XML Treatment for Inocybe tumericolor

XML Treatment for Inocybe subaeruginascens

## Additional information

### Artificial Intelligence (AI) use

The authors accept full responsibility for the content of the manuscript, including the disclosure of any use of AI.

No AI tools were used in the preparation of this manuscript.

### Author ORCIDs

J.-Q. Liang https://orcid.org/0000-0001-9493-7651

X. Chen https://orcid.org/0009-0008-1535-6911

T.-Z. Liu https://orcid.org/0000-0003-1751-2195

Y.-Z. Zhang https://orcid.org/0000-0001-7300-2400

H.-J. Li https://orcid.org/0000-0002-7227-4479

Y.-G. Fan https://orcid.org/0000-0002-3028-9752

F. Xu https://orcid.org/0000-0001-9219-1235

### Data availability

All sequence data generated for this study can be accessed via GenBank: https://www.ncbi.nlm.nih.gov/genbank/.

## References

[B1] Bandini D, Oertel B, Eberhardt U (2023) Even more fibre-caps (4): Fourteen new species of the family *Inocybaceae*. Mycologia Bavarica 23: 1–50.

[B2] Bau T, Fan YG (2018) Three new species of *Inocybe* sect. *Rimosae* from China. Mycosystema 37: 693–702. 10.13346/j.mycosystema.180033

[B3] Bau T, Bao HY, Yu LI (2014) A revised checklist of poisonous mushrooms in China. Mycosystema 33: 517–548.

[B4] Bau T, Li HJ, Bao HY, Li Y (2024) A revised checklist of poisonous mushrooms in China. Journal of Fungal Research 22(4): 301–321. 10.13341/j.jfr.2024.0010

[B5] Bhunjun CS, Niskanen T, Suwannarach N, Wannathes N, Chen Y-J, McKenzie EHC, Maharachchikumbura SSN, Buyck B, Zhao C-L, Fan Y-G, Zhang J-Y, Dissanayake AJ, Marasinghe DS, Jayawardena RS, Kumla J, Padamsee M, Chen Y-Y, Liimatainen K, Ammirati JF, Phukhamsakda C, Liu J-K, Phonrob W, Randrianjohany É, Hongsanan S, Cheewangkoon R, Bundhun D, Khuna S, Yu W-J, Deng L-S, Lu Y-Z, Hyde KD, Lumyong S (2022) The numbers of fungi: are the most speciose genera truly diverse? Fungal Diversity 114: 387–462. 10.1007/s13225-022-00501-4

[B6] Chen X, Yu WJ, Bau T, Matheny PB, Horak E, Liu Y, Qin LW, Tang LP, Ge YP, Liu TZ, Fan YG (2024) Contributions to the *Inocybe umbratica–paludinella* (*Agaricales*) group in China: Taxonomy, species diversity, and molecular phylogeny. Journal of Fungi 10(12): e893. 10.3390/jof10120893 PMC11677058 39728389

[B7] Chen ZH, Yang ZL, Bau T, Li TH (2016) Poisonous mushroom: Recognition and poisoning treatment. Science Press, Beijing.

[B8] Deng LS, Kang R, Zeng NK, Yu WJ, Chang C, Xu F, Deng WQ, Qi LL, Zhou YL, Fan YG (2021) Two new *Inosperma (Inocybaceae)* species with unexpected muscarine contents from tropical China. MycoKeys 85: 87–108. 10.3897/mycokeys.85.71957 PMC8695569 35035255

[B9] Dinis-Oliveira RJ (2017) Metabolism of psilocybin and psilocin: clinical and forensic toxicological relevance. Drug Metabolism Reviews 49: 84–91. 10.1080/03602532.2016.1278228 28074670

[B10] EU (2021) Commission Implementing Regulation (EU) 2021/808 of 22 March 2021 on the performance of analytical methods for residues of pharmacologically active substances used in food-producing animals and on the interpretation of results as well as on the methods to be used for sampling and repealing Decisions 2002/657/EC and 98/179/EC [EU/2021/808]. http://data.europa.eu/eli/reg_impl/2021/808/2021-06-10 [Accessed 12 December 2023]

[B11] Fan YG, Bau T (2010) A revised checklist of the genus *Inocybe* (Fr.) Fr. in China. Journal of Fungal Research 8: 189–193.

[B12] Fan YG, Bau T (2013) Two striking *Inocybe* species from Yunnan Province, China. Mycotaxon 123: 169–181. 10.5248/123.169

[B13] Fan YG, Bau T (2014a) *Inocybe hainanensis*, a new lilac-stiped species from tropical China. Mycosystema 33: 954–960.

[B14] Fan YG, Bau T (2014b) *Inocybe miyiensis*, a new two-spored species in section *Marginatae* from China. Nova Hedwigia 98(1–2): 179–185. 10.1127/0029-5035/2013/0135

[B15] Fan YG, Bau T (2017) Three newly recorded species of *Inocybe subg. Inocybe* in China. Mycosystema 36: 251–259. 10.13346/j.mycosystema.150274

[B16] Fan YG, Bau T (2020) Two new smooth-spored species of *Inocybe* (*Inocybaceae*, *Agaricales*) from Gansu Province, northwestern China. Mycosystema 39: 1694–1705. 10.13346/j.mycosystema.200210

[B17] Fan YG, Bau T, Kobayashi T (2013) Newly recorded species of *Inocybe* collected from Liaoning and Inner Mongolia. Mycosystema 32: 302–308.

[B18] Fan YG, Wu RH, Bau T (2018) Two new species and eight newly recorded species of subg. *Inocybe* from China. Journal of Fungal Research 16: 70–83. 10.13341/j.jfr.2018.1211

[B19] Fries E (1863) Monographia Hymenomycetum Sueciae. CA LeZer, Uppsala.

[B20] Gao JL, Ge YP, Matheny PB, He PM, Wu XP, Bau T, Yu WJ, Fan YG (2025) A phylogeny of the *Inocybe alienospora* group (*Agaricales*) with emphasis on seven new species from China and emendation of sect. *Leptocybe*. Mycology 16(2): 812–855. 10.1080/21501203.2024.2380069 PMC12096670 40415917

[B21] Griffiths RR, Richards WA, McCann U, Jesse R (2006) Psilocybin can occasion mystical-type experiences having substantial and sustained personal meaning and spiritual significance. Psychopharmacology 187: 268–283. 10.1007/s00213-006-0457-5 16826400

[B22] Griffiths RR, Richards WA, Johnson MW, McCann UD, Jesse R (2008) Mystical-type experiences occasioned by psilocybin mediate the attribution of personal meaning and spiritual significance 14 months later. Journal of Psychopharmacol 22: 621–632. 10.1177/0269881108094300 PMC3050654 18593735

[B23] Griffiths RR, Johnson MW, Richards WA, Richards BD, McCann UD, Jesse R (2011) Psilocybin occasioned mystical-type experiences: immediate and persisting doserelated effects. Psychopharmacology (Berl) 218: 649–665. 10.1007/s00213-011-2358-5 PMC3308357 21674151

[B24] Hall TA (1999) BioEdit: a user-friendly biological sequence alignment editor and analysis program for Windows 95/98/NT. Nucleic Acids Symposium Series 41: 95–98.

[B25] Hu JH, Yu WJ, Deng LS, Fan YG, Bau T, Tang LP, Lin WF, Deng CY (2023) The detection of major clades and new species of *Mallocybe* (*Inocybaceae*, *Agaricales*) from China with elongate cheilocystidia. Mycological Progress 22: 15. 10.1007/s11557-022-01854-5

[B26] Katoh K, Rozewicki J, Yamada KD (2019) MAFFT online service: multiple sequence alignment, interactive sequence choice and visualization. Briefings in Bioinformatics 20: 1160–1166. 10.1093/bib/bbx108 PMC6781576 28968734

[B27] Kornerup A, Wanscher JH (1978) The Methuen Handbook of Colour, 3^rd^ edn. Eyre Methuen Ltd., Reprint, London.

[B28] Kosentka P, Sprague S, Ryberg M, Gartz J, May A, Campagna S, Matheny P (2013) Evolution of the toxins muscarine and psilocybin in a family of mushroom-forming fungi. PLOS ONE 8: e64646. 10.1371/journal.pone.0064646 PMC3662758 23717644

[B29] Kuyper TW (1986) A revision of the genus *Inocybe* in Europe I. Subgenus *Inosperma* and the smooth-spored species of subgenus *Inocybe*. Persoonia (Supplement 3): 1–247.

[B30] Li HJ, Zhang HS, Zhang YZ, Zhang KP, Zhou J, Yin Y, Jiang SF, Ma P, He Q, Zhang YT, Wen K, Yuan Y, Lang N, Lu JJ, Sun CY (2020) Mushroom poisoning outbreaks—China, 2019. China CDC Weekly 2: 19–24. 10.46234/ccdcw2020.005 PMC8392910 34594654

[B31] Li HJ, Zhang HS, Zhang YZ, Zhou J, Yin Y, He Q, Jiang SF, Ma PB, Zhang YT, Wen K, Yuan Y, Lang N, Cheng BW, Lu JJ, Sun CY (2021a) Mushroom poisoning outbreaks—China, 2020. China CDC Weekly 3: 41–45. 10.46234/ccdcw2021.014 PMC8392932 34594953

[B32] Li HJ, Zhang HS, Zhang YZ, Zhou J, Yin Y, He Q, Jiang SF, Ma PB, Zhang YT, Yuan Y, Lang N, Cheng BW, Wang M, Sun CY (2022a) Mushroom poisoning outbreaks—China, 2021. China CDC Weekly 4: 35–40. 10.46234/ccdcw2022.010 PMC8796718 35586461

[B33] Li HJ, Zhang YZ, Zhang HS, Zhou J, Liang JQ, Yin Y, He Q, Jiang SF, Zhang YT, Yuan Y, Lang N, Cheng BW, Wang M, Sun CY (2023) Mushroom poisoning outbreaks—China, 2022. China CDC Weekly 5: 45–50. 10.46234/ccdcw2023.009 PMC9902756 36776462

[B34] Li HJ, Zhang YZ, Zhang HS, Zhou J, Chen ZH, Liang JQ, Yin Y, He Q, Jiang SF, Zhang YT, Yuan Y, Lang N, Cheng BW, Zhong JJ, Li ZF, Sun CY (2024) Mushroom poisoning outbreaks—China, 2023. China CDC Weekly 6(4): 64–68. 10.46234/ccdcw2024.014 PMC10832152 38313818

[B35] Li HJ, Zhang YZ, Zhang HS, Zhou J, Li ZF, Yin Y, He Q, Jiang SF, Zhang YT, Yuan Y, Lang N, Cheng BW, Zhong JJ, Yuan MX, Liu ZY, Sun CY (2025) Mushroom Poisoning Outbreaks—China, 2024. China CDC Weekly 7(19): 645–649. 10.46234/ccdcw2025.106 PMC12075492 40376178

[B36] Li SN, Xu F, Jiang M, Liu F, Wu F, Zhang P, Fan YG, Chen ZH (2021b) Two new toxic yellow *Inocybe* species from China: morphological characteristics, phylogenetic analyses and toxin detection. MycoKeys 81: 185. 10.3897/mycokeys.81.68485 PMC8355026 34385885

[B37] Li SN, Xu F, Long P, Liu F, Zhang P, Fan YG, Chen ZH (2022b) Five new species of *Inosperma* from China: Morphological characteristics, phylogenetic analyses, and toxin detection. Frontiers in Microbiology 13: 1021583. 10.3389/fmicb.2022.1021583 PMC9659589 36386664

[B38] Ludwig E (2017) Pilzkompendium. Band 4. *Cortinariaceae* (*Galerina*, *Hebeloma*, *Inocybe*, *Phaeogalera*, *Cortinarius* Teil I mit den Untergattungen *Cortinarius*, *Dermocybe*, *Leprocybe*, *Phlegmacium*). Fungicon-Verlag, Berlin.

[B39] Mao N, Xu YY, Zhao TY, Lv JC, Fan L (2022) New Species of *Mallocybe* and *Pseudosperma* from North China. Journal of Fungi 8: 256. 10.3390/jof8030256 PMC8949120 35330258

[B40] Mao XL (2006) Poisonous mushrooms and their toxins in China. Mycosystema 25: 345–363.

[B41] Matheny PB (2005) Improving phylogenetic inference of mushrooms with RPB1 and RPB2 nucleotide sequences (*Inocybe*, *Agaricales*). Molecular Phylogenetics and Evolution 35: 1–20. 10.1016/j.ympev.2004.11.014 15737578

[B42] Matheny PB, Kudzma LV (2019) New species of *Inocybe (Inocybaceae)* from eastern north America. The Journal of the Torrey Botanical Society 146(3): 213–235. 10.3159/TORREY-D-18-00060.1

[B43] Matheny PB, Hobbs AM, Esteve-Raventós F (2020) Genera of *Inocybaceae*: New skin for the old ceremony. Mycologia 112: 83–120. 10.1080/00275514.2019.1668906 31846596

[B44] Nylander JAA (2004) Bayesian phylogenetics and the evolution of gall wasps. Acta Universitatis Upsaliensis, Uppsala, 43 pp.

[B45] Patocka J, Wu R, Nepovimova E, Valis M, Wu W, Kuca K (2021) Chemistry and toxicology of major bioactive substances in *Inocybe* mushrooms. International Journal of Molecular Sciences 22: 2218. 10.3390/ijms22042218 PMC7926736 33672330

[B46] Ronquist F, Teslenko M, Van Der Mark P, Ayres DL, Darling A, Höhna S, Larget B, Liu L, Suchard MA, Huelsenbeck JP (2012) MrBayes 3.2: efficient Bayesian phylogenetic inference and model choice across a large model space. Systematic Biology 61: 539–542. 10.1093/sysbio/sys029 PMC3329765 22357727

[B47] Tamura K, Peterson D, Peterson N, Stecher G, Nei M, Kumar S (2011) MEGA5: molecular evolutionary genetics analysis using maximum likelihood, evolutionary distance, and maximum parsimony methods. Molecular Biology and Evolution 28: 2731–2739. 10.1093/molbev/msr121 PMC3203626 21546353

[B48] Tang LX, Zhang YZ, Liang JQ, Jiang SF, Si J, Li HJ (2023) *Pyrofomes lagerstroemiae* (*Polyporaceae*, *Basidiomycota*), a new species from Hubei Province, Central China. Phytotaxa 583: 191–198. 10.11646/phytotaxa.583.2.7

[B49] Trifinopoulos J, Nguyen LT, von Haeseler A, Minh BQ (2016) W-IQ-TREE: a fast online phylogenetic tool for maximum likelihood analysis. Nucleic Acids Research 44: W232–W235. 10.1093/nar/gkw256 PMC4987875 27084950

[B50] Vilgalys R, Hester M (1990) Rapid genetic identification and mapping of enzymatically amplified ribosomal DNA from several *Cryptococcus* species. Journal of Bacteriology 172: 4238–4246. 10.1128/jb.172.8.4238-4246.1990 PMC213247 2376561

[B51] Wang SN, Hu YP, Chen JL, Qi LL, Zeng H, Ding H, Huo GH, Zhang LP, Chen FS, Yan JQ (2021) First record of the rare genus *Typhrasa* (*Psathyrellaceae*, *Agaricales*) from China with description of two new species. MycoKeys 79: 119–128. 10.3897/mycokeys.79.63700 PMC8087613 33958951

[B52] White J, Weinstein S, Haro L, Bedry R, Schaper A, Rumack B, Zilker T (2019) Mushroom poisoning: A proposed new clinical classification. Toxicon 157: 53–65. 10.1016/j.toxicon.2018.11.007 30439442

[B53] White TJ, Bruns T, Lee S, Taylor J (1990) Amplification and direct sequencing of fungal ribosomal RNA genes for phylogenetics. In: Innis MA, Gelfand DH, Sninsky JJ, White TJ (Eds) PCR Protocols: A Guide to Methods and Applications. Academic Press, New York, 315–322. 10.1016/B978-0-12-372180-8.50042-1

[B54] Wu F, Zhou LW, Yang ZL, Bau T, Li TH, Dai YC (2019) Resource diversity of Chinese macrofungi: edible, medicinal and poisonous species. Fungal Diversity 98: 1–76. 10.1007/s13225-019-00432-7

[B55] Xu F, Zhang YZ, Zhang YH, Guan GY, Zhang KP, Li HJ, Wang JJ (2020) Mushroom poisoning from *Inocybe serotina*: A case report from Ningxia, northwest China with exact species identification and muscarine detection. Toxicon 179: 72–75. 10.1016/j.toxicon.2020.03.003 32345453

[B56] Yu WJ, Chang C, Qin LW, Zeng NK, Wang SX, Fan YG (2020) *Pseudosperma citrinostipes* (*Inocybaceae*), a new species associated with *Keteleeria* from southwestern China. Phytotaxa 450(1): 8–16. 10.11646/phytotaxa.450.1.2

[B57] Zhang YZ, Yan YY, Li HJ, Fan YG, Xu F (2022) Toxin screening of *Pseudosperma umbrinellum* (*Agaricals*, *Basidiomycota*): First report of phalloidin in *Inocybaceae* mushroom. Toxicon 217: 155–161. 10.1016/j.toxicon.2022.08.005 35998714

[B58] Zhao LN, Yu WJ, Deng LS, Hu JH, Ge YP, Zeng NK, Fan YG (2022) Phylogenetic analyses, morphological studies, and muscarine detection reveal two new toxic *Pseudosperma* (*Inocybaceae*, *Agaricales*) species from tropical China. Mycological Progress 21: 75. 10.1007/s11557-022-01822-z

[B59] Zhu A-H, Xu Y-R, Ma H-X, Yu W-J (2025) Three nodulose-spored *Inocybe* (*Agaricales*, *Basidiomycota*) species discovered from Motuo, Southwest China. MycoKeys 122: 99–121. 10.3897/mycokeys.122.163942.figure1 PMC12447083 40980477

